# Nemertean Toxin Genes Revealed through Transcriptome Sequencing

**DOI:** 10.1093/gbe/evu258

**Published:** 2014-11-27

**Authors:** Nathan V. Whelan, Kevin M. Kocot, Scott R. Santos, Kenneth M. Halanych

**Affiliations:** ^1^Department of Biological Sciences, Molette Biology Laboratory for Environmental and Climate Change Studies, Auburn University, Auburn, Alabama; ^2^Present address: School of Biological Sciences, University of Queensland, Brisbane, QLD, Australia

**Keywords:** Nemertea, ribbon worm, toxin, cytotoxin, hepatotoxin, transcriptome

## Abstract

Nemerteans are one of few animal groups that have evolved the ability to utilize toxins for both defense and subduing prey, but little is known about specific nemertean toxins. In particular, no study has identified specific toxin genes even though peptide toxins are known from some nemertean species. Information about toxin genes is needed to better understand evolution of toxins across animals and possibly provide novel targets for pharmaceutical and industrial applications. We sequenced and annotated transcriptomes of two free-living and one commensal nemertean and annotated an additional six publicly available nemertean transcriptomes to identify putative toxin genes. Approximately 63–74% of predicted open reading frames in each transcriptome were annotated with gene names, and all species had similar percentages of transcripts annotated with each higher-level GO term. Every nemertean analyzed possessed genes with high sequence similarities to known animal toxins including those from stonefish, cephalopods, and sea anemones. One toxin-like gene found in all nemerteans analyzed had high sequence similarity to Plancitoxin-1, a DNase II hepatotoxin that may function well at low pH, which suggests that the acidic body walls of some nemerteans could work to enhance the efficacy of protein toxins. The highest number of toxin-like genes found in any one species was seven and the lowest was three. The diversity of toxin-like nemertean genes found here is greater than previously documented, and these animals are likely an ideal system for exploring toxin evolution and industrial applications of toxins.

## Introduction

Many animal lineages have independently evolved the use of toxins to enhance their fitness (Fry, Roelants, Champagne, et al. 2009; Fry, Roelants and Norman, 2009; Casewell et al. 2013; Undheim et al. 2014), and humans have capitalized on a multitude of these chemicals for pharmaceuticals and other industrial applications (Kalman et al. 1998; Lewis and Garcia 2003; King 2011; Upadhyay et al. 2013). Depending on the species, toxins utilized by animals may be obtained through ingestion of other organisms (Paul and Van Alstyne 1988; Paul and Pennings 1991), produced by bacterial symbionts (Carroll et al. 2003) or synthesized by the animal itself (Casewell et al. 2013). In general, toxins are employed for either prey capture (e.g., cone snails [Olivera et al. 1985], venomous snakes [Lee 1979], cnidarians [Turk and Kem 2009]) or defense from predators (e.g., poison dart frogs [Daly et al. 1978; Saporito et al. 2012] and rough skinned newts [Brodie 1968]). Toxin genes are particularly interesting from an evolutionary perspective as toxic proteins with similar form and function show convergent evolution in different animal groups (reviewed by Fry, Roelants, Champagne, et al. 2009; Casewell et al. 2013). Despite the potential utility of toxins for biomedical and other applications, precise chemical properties of many toxins and the breadth of toxins produced across life, remain unknown. This lack of information limits our understanding of toxin evolution as well as evolution of predation and defense mechanisms (Brodie and Brodie 1990; Jost et al. 2008).

One group that has received relatively little attention in regards to toxins is Nemertea, or ribbon worms, with multiple recent reviews on animal toxins having failed to mention this group (Fry, Roelants, Champagne, et al. 2009; Fry, Roelants and Norman 2009; Casewell et al. 2013). Nemertea contains ∼1,500 species of mostly marine organisms (Gibson 1995; Kajihara et al. 2008), and they are perhaps best known for a long, eversible proboscis, used in prey capture (Stricker and Cloney 1981; McDermott and Roe 1985; Thiel and Kruse 2001). Although the proboscis is typically used to capture and immobilize prey, nemerteans can also employ toxins to paralyze or kill prey (McDermott and Roe 1985; Asakawa et al. 2013). Nemerteans also utilize toxins for predator defense (Bacq 1937; Kem 1971, 1976; Kem et al. 1971; McClintock and Baker 1997; Carroll et al. 2003) by secreting toxins from cells in their epidermis (Norenburg 1985). For example, toxins in the body wall of *Parbolasia corrugatus* provide defense from potential predators in Antarctic waters (McClintock and Baker 1997). Nemerteans can be prominent members of marine benthic (Gibson 1983; McClintock and Baker 1997) and pelagic communities (Roe and Norenburg 1999), but many species appear to have few or no predators, apparently because of the effectiveness of their acidic and/or chemically defended body walls (Ling 1970; Dayton et al. 1974; Ferraris 1979; Ali et al. 1990; Heine et al. 1991; McClintock and Baker 1997). Despite toxins being an integral part of nemertean biology, few have been characterized.

Information about nemertean toxin genes and their products is needed to understand how nemerteans work to enhance their predatory and defensive effectiveness. To date, the limited studies on nemertean toxin genes and their products have mostly focused on nonpeptides such as tetrodotoxin (Ali et al. 1990; Carroll et al. 2003; Tanu et al. 2004) and anabaseine (Kem 1971; Kem et al. 1971). Thus far, only three peptide toxins have been described from nemerteans: cytotoxin A-III in *Cerebratulus lacteus* and *P. corrugatus* and neurotoxin B-II and neurotoxin B-IV in *C. lacteus* (Kem 1976, 1994; Blumenthal et al. 1981; Barnham et al. 1997; Berne et al. 2003). Overall, genomic underpinnings of most nemertean toxins and whether the aforementioned peptide toxins are found in other nemertean species have not been assessed. As such, whether nemerteans primarily obtain toxins from the environment and/or symbionts (e.g., tetrodotoxin from bacteria; Carroll et al. 2003) or if they possess an unrecognized diversity of toxin genes is unclear.

Transcriptome sequencing can be a critical tool for understanding the ecology and evolution of understudied animals and provides resources for future scientific study. Furthermore, advances in sequencing technologies and bioinformatics allow for comprehensive characterization of animal transcriptomes and exploration of toxin genes with relative ease (Garber et al. 2011; Hass et al. 2013). In this study, we searched transcriptomes of nine nemerteans for toxin genes. We sequenced and characterized transcriptomes of two free-living nemerteans, *Paranemertes peregrina* and *Tubulanus polymorphus*, and one commensal nemertean, *Malacobdella grossa*. We also analyzed publicly available transcriptome data of *Cephalothrix hongkongiensis*, *Cephalothrix linearis*, *Cerebratulus marginatus*, two *Lineus* species, and *Ramphogordius lacteus* to further characterize nemertean transcriptomes and survey toxin gene diversity across the group. We consider any gene coding for a protein with high similarity to known toxins in other animal groups as a putative toxin gene. *Malscobdella grossa*, which lives in the mantle cavity of bivalves, was hypothesized to have fewer toxin genes than other species to potentially minimize harm to its host. Similarities between nemertean genes and toxins from other animals add to the growing body of evidence supporting convergent evolution in animal-derived peptide toxins.

## Materials and Methods

### Specimen Sampling and Sequencing

*Malacobdella grossa* was collected off Rhode Island by a commercial vessel harvesting the bivalve *Arctica islandica* as part of the study by Dahlgren et al. (2000). *Paranemertes peregrina* and *T. polymorphus* were collected in False Bay, San Juan Island, Washington. RNA extraction, complimentary DNA (cDNA) library preparation, and Illumina sequencing generally followed the methods of Weigert et al. (2014). In brief, we extracted RNA from *M. grossa* and *Para. peregrina* using whole animals and from *T. polymorphus* using the anterior three quarters of the specimen with TRIzol (Invitrogen). RNA was purified with the Qiagen RNeasy kit (Valencia, CA) using on-column DNAse digestion. cDNA libraries were constructed with the SMART cDNA library construction kit (Clontech Laboratories, Mountain View, CA) following the manufacturer’s protocol except that the provided 3' oligo was replaced with the Cap-Trsa-CV oligo as per Meyer et al. (2009). Full-length cDNA was then amplified using the Advantage 2 PCR system (Clontech) with a minimum number of PCR cycles (i.e., 17–21) and sent to HudsonAlpha Institute for Biotechnology (Huntsville, AL) for library preparation and sequencing on an Illumina HiSeq 2000 using 2 × 100 bp paired-end (PE) chemistry. Data for five other nemerteans were retrieved from NCBI (table 1; Riesgo et al. 2012).

**Table 1:** Assembly Statistics

Species	GenBank Number	Sequencing Platform	Raw reads	N50	GC%	Mean Contig Length	Number of Contigs	Longest contig	% Partial CEGMA Genes	% Complete CEGMA Genes
*M. grossa*	SRX731465	HiSeq 2x100	83,045,507	1720	43.8	1047.06	109,120	15,014	99.19	93.55
*Para. peregrina*	SRX731466	HiSeq 2x100	86,271,698	1493	45.6	869.65	99,203	25,470	93.95	86.69
*T. polymorphus*	SRX732127	HiSeq 2x100	39,262,732	1492	39.3	916.39	79,313	13,710	98.79	88.31
*Cep. hongkongiensis*	SRX205320	Genome Analyzer II 2x100	26,112,259	471	36.9	431.00	122,233	4,383	65.32	16.53
*Cep. linearis*	SRX524866–SRX534868	Genome Analyzer IIx 2x75	12,062,451	1241	37.4	738.21	67,317	14,570	97.58	86.69
*C. marginatus*	SRX205323	Genome Analyzer II 2x100	26,688,280	515	41.6	458.84	194,257	8,534	83.47	28.23
*L. longissimus*	SRX565176–SRX565181	HiSeq 1x100	95,576,010	2292	43.0	1186.57	79,452	25,184	98.79	93.55
*L. ruber*	SRXX565182–SRX565183	HiSeq 1x100	59,763,351	2105	43.6	1082.25	156,014	26,007	98.39	92.74
*R. lacteus*	SRX565174, SRX565175	HiSeq 1x100	25,452,210	3246	43.1	1539.37	96,066	23,116	98.79	91.94

### Transcriptome Assembly

Transcriptome read quality was assessed with the FASTX toolkit (Gordon 2011). Given overall high read quality, sequences were not filtered prior to assembly. All raw data underwent digital normalization using the python script normalize-by-median.py (Brown et al. 2012) with a k-mer size of 20, a desired coverage (i.e., cutoff) of 30, and four hash tables with a lower bound of 2.5 × 10^9^. Trinity version November 2013 (Grabherr et al. 2011; Haas et al. 2013) was utilized for transcriptome assembly of normalized reads for each species with a k-mer size of 25. Raw reads were assembled as PE data except for the two *Lineus* species and *R. lacteus*, for which only single-end reads were available.

### Assessment of Assembly Quality

A rarefaction curve (Sanders 1968) of assembly statistics was used to evaluate quality and completeness of assemblies from the newly sequenced species because a nemertean reference genome was not available for comparison. Specifically, we removed 10–70% of PE sequences from the end of raw read files to produce datasets with reduced numbers of reads. These subsampled datasets were assembled as above, and N50 and total number of contigs for each assembly was plotted. A plateau on the rarefaction curve would indicate that adding more sequence data would not considerably change the characteristics of the transcriptome assembly. We further measured completeness of each transcriptome assembly with CEGMA 2.4 (Parra et al. 2007), which determines how many of 248 core eukaryotic genes were present in each transcriptome. Core genes annotated by CEGMA are ones that are highly conserved and chosen from the eukaryotic orthologous groups database (Tatusov et al. 2003). An advantage to an approach like CEGMA is that potential differences in which genes are present among assemblies, as a result of expression differences in various tissue types used for cDNA library preparation, is minimized because housekeeping genes should be expressed in virtually all cells.

### Transcriptome Annotation

Annotation of each assembled transcriptome was done with the Trinotate annotation suite (http://trinityrnaseq.sourceforge.net/annotation/Trinotate.html, last accessed April 13, 2014). In brief, TransDecoder (Haas et al. 2013) was first used to predict open reading frames (ORFs) of at least 300 bp. If multiple, overlapping ORFs were present in the same contig, only the longest ORF was retained. In contrast, if multiple but nonoverlapping 300 bp ORFs were identified, all were retained. Thus, two or more ORFs could originate from the same transcript (i.e., ORFs on both forward and reverse strands and/or multiple ORFs on the same strand for long contigs). Untranslated transcripts and translated ORFs were then queried against the Swiss-Prot database (UniProt Consortium 2014) using Basic Local Alignment Search Tool x (BLASTx) and BLASTp, respectively (Altschul et al. 1997), with annotation coming from the best BLAST hit and associated Gene Ontology (GO) terms (Ashburner et al. 2000). Trinotate then used HMMER 3.1 tool hmmscan (Eddy 2001; Finn et al. 2011) and the Pfam-A database (Punta et al. 2014) to annotate protein domains for each predicted protein sequence. Trinotate results were populated into a SQLite database and placed into a tab delimitated file with scripts provided in the Trinotate package and a custom wrapper (available from http://github.com/halocaridina/bioinformatic-scripts, last accessed May 15, 2014). To roughly characterize the protein composition of each nemertean transcriptome, a custom python script (available from http://github.com/NathanWhelan, last accessed May 15, 2014) was used to place GO terms for each UniProt annotated transcript from Trinotate into Web Gene Ontology Annotation Plotting (WEGO) format, and annotated GO terms were visualized using the WEGO web service (Ye et al. 2006).

Toxin genes were identified based on sequence similarity to previously characterized animal toxins genes under the assumption that sequence similarity is generally indicative of function (Gabaldón and Huynen 2004). Putative toxin genes were initially distinguished if top BLASTx and/or BLASTp hits in the Trinotate output were a previously characterized eukaryotic toxin gene (as defined by Swiss-Prot or presence of a Pfam domain with “toxin” in the description). Genes of putative viral or bacterial origin were discarded, which eliminated putative toxin genes that may have been from bacterial endosymbionts or from horizontal gene transfer events. Amino acid sequences of remaining transcripts were then manually searched against the NCBI nonredundant GenBank database (nr) and the Pfam protein domain database using the HMMER 3.1 tool phmmer (Eddy 2001; Finn et al. 2011). Annotated sequences initially identified as a toxin gene by Swiss-Prot were further considered a toxin gene if either 1) cross validation produced a significant hit for a toxin domain family in Pfam or 2) if the best annotated hit from the nr database was labeled a toxin gene. In some instances, a toxin as identified by Trinotate and Swiss-Prot did not have a toxin gene as highest hit against the nr database using phmmer, nor did these transcripts possess a toxin protein domain according to Pfam-A; such transcripts were not further considered as they were potential false positives. Toxin genes passing the above filters were then reciprocally queried against the other transcriptomes to identify putative orthologs via BLAST searches. We also queried previously identified nemertean peptide toxins (i.e., Cytotoxin A-III, Neurotoxin B-II, and Neurotoxin B-IV; Kem 1976; Blumenthal et al. 1981) against all transcriptomes with a tBLASTn search.

### Gene Tree Reconstruction

Gene trees were inferred for stonefish toxin (SNTX)-like and Plancitoxin-1-like genes because they were found in all nine nemerteans. Putative SNTX and Plancitoxin genes were translated with TransDecoder (Haas et al. 2013) using default settings. Redundant nemertean protein sequences were then removed from the dataset. The SNTX dataset of von Reumont (2014) was added to the nemertean SNTX-like genes. Putative Plancitoxin-1 genes and nontoxic DNase II genes were retrieved from UniProt and GenBank (supplementary figs. S1 and S2, Supplementary Material online). Alignments were done in MAFFT 3 with the E-INS-i algorithm (Katoh and Standley 2013). The appropriate model of protein evolution was selected for each gene using ProtTest 3.4 (LG + F + Γ for both genes; Darriba et al. 2011). RAxML 8 (Stamatakis 2014) was used to infer maximum likelihood gene trees, and 1,000 nonparametric bootstrap replicates were performed to assess nodal support. Trees were rooted with nontoxic homologs.

## Results

### Transcriptome Assembly and Characterization

Average read quality score was above 30 for all taxa except *Para. peregrina*, which had an average quality score of 28.7. Assemblies from Trinity (figshare, doi:10.6084/m9.figshare.1203580) possessed an average of 111,441.7 (±40,859 SD) contigs per taxon—full assembly and sequencing statistics are presented in table 1. Rarefaction curves of the three taxa sequenced here showed a steady increase in N50 and number of contigs as the amount of total data increased (supplementary fig. S1, Supplementary Material online), with a flat plateau not being reached for either measurement. However, the overall trend strongly suggested that increasing the number of reads would result in diminishing returns regarding the total number of transcripts recovered. Furthermore, the high percentage of CEGMA core genes present (>85% of core genes had complete sequences; >93% of core genes were partially sequenced) in each full assembly of newly sequenced transcriptome (table 1) suggests that each assembly possesses a representative snapshot of the mRNA content expected from a typical metazoan cell. In contrast, assemblies from *C. marginatus* and *Cep. hongkongiensis* possessed much lower percentages of CEGMA core genes (<30% core genes had complete sequences; table 1).

**Fig. 1.—:**
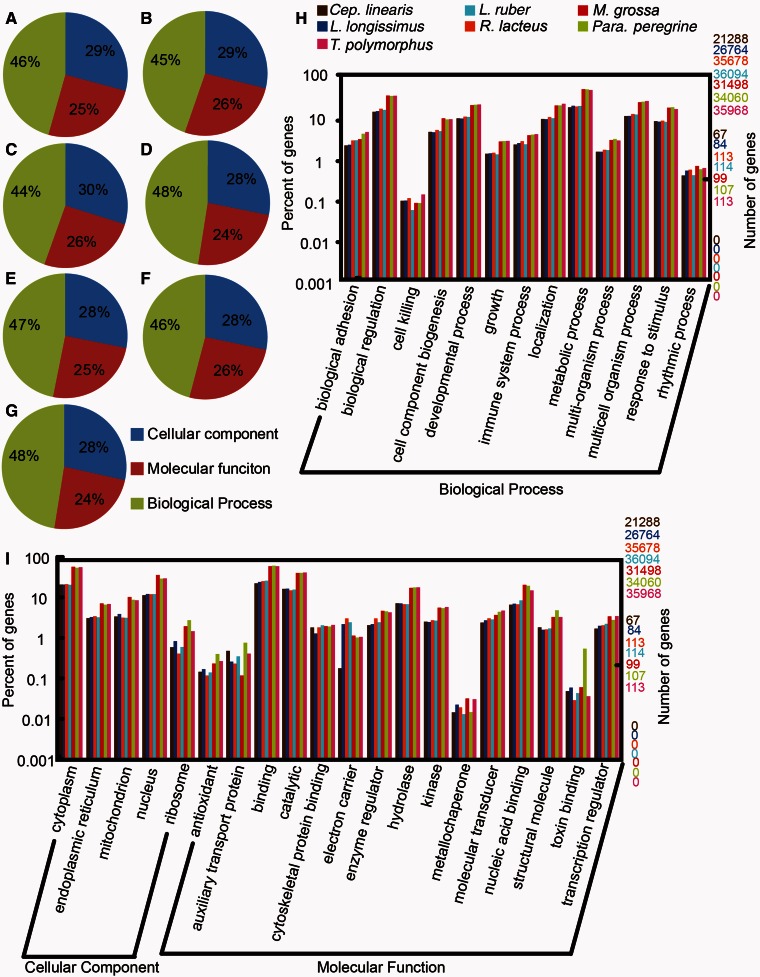
*A–G* Pie charts with percentages of the higher-level GO terms for each species except *Cep. hongkongeinsis* and *C. marginatus* (see Riesgo et al. 2012). (*A*) *M. grossa*, (*B*) *Para. peregrine*, (*C*) *T. polymorphus*, (*D*) *Cep. linearis*, (*E*) *L. longissimus*, (*F*) *L. ruber*, (*G*) *R. lacteus*. (*H*) Bar chart of genes annotated with select “Biological Process” lower-level GO terms. (*I*) Bar chart of genes annotated with select “Cellular Component” and “Molecular Function” lower-level GO terms. Bar plot colors are referenced in (*H*).

Annotation reports from Trinotate for each transcriptome can be found on figshare (doi:10.6084/m9.figshare.1203580). In total, 18.2–46.2% of putative transcripts had predicted ORFs and 63.1–91.7% of inferred ORFs were annotated with a significant BLASTp hit (table 2). Of these annotated transcripts, most had multiple GO terms. For all species, the highest percentage of genes was annotated as biological process (44–48%). Lower percentages of genes were annotated as either cellular component (28–30%) or molecular function (24–26%) (fig. 1*A*–*G*; data for *C. marginatus* and *Cep. hongkongiensis* not shown, but can be seen in Riesgo et al. 2012). Overall, lower-level GO term annotations were similar across all nine species, but *M. grossa*, *Para. peregrina*, and *T. polymorphus* typically had more genes annotated for any given term than other species (fig. 1*H*). Auxiliary transport protein, metabolic chaperone, and toxin binding are notable exceptions to this pattern (fig. 1*H*).

**Table 2:** Annotation Statistics

Species	Number (%) of transcripts annotated with BLASTn	Number (%) of transcripts with predicted ORF	Number (%) of ORFs annotated with BLASTp
*M. grossa*	33,964 (41.8)	40,421 (37.0)	30,072 (74.4)
*Para. peregrina*	36,452 (35.7)	40,530 (40.9)	28,460 (70.2)
*T. polymorphus*	38,364 (34.3)	51,660 (46.2)	33,904 (65.6)
*Cep. hongkongiensis*	39,525 (32.2)	37,688 (30.7)	24,329 (64.5)
*Cep. linearis*	22,328 (32.9)	25,652 (37.8)	18,391 (71.7)
*C. marginatus*	32,011 (16.4)	35,451 (18.2)	22,357 (63.1)
*L. longissimus*	29,048 (35.4)	35,714 (47.8)	24,852 (70.0)
*L. ruber*	39,770 (24.6)	47,288 (47.2)	33,468 (70.8)
*R. lacteus*	38,771 (38.7)	52,422 (32.4)	33,809 (64.5)

### Nemertean Toxin Genes

Putative toxin genes were recovered from all nemertean transcriptomes examined here (table 3), and the number of such genes ranged from three in the commensal *M. grossa* to seven in *C. marginatus*, *Lineus ruber*, and both *Cephalothrix* species. In some cases, multiple transcripts of the same toxin-like gene were found in each assembly. The two most common putative toxin genes were Plancitoxin-1-like (Shiomi et al. 2004) and stonefish-like toxin genes (SNTX/VTX family; Ueda et al. 2006). All Plancitoxin-1-like genes possessed a DNase II protein domain. Six other types of toxin-like genes were identified, but less frequently, among species analyzed. Putative toxin genes with at least two ShK protein domains downstream of cysteine rich secretory domains (CAP; Gibbs et al. 2008) were found in *M. grossa*, *Para. peregrina*, and *Cep. linearis*. Natterin-4-, Echotoxin-2-, and SE-cephalotoxin-like genes were recovered in four, five, and six of the nemerteans analyzed here, respectively (table 3). Toxin-like genes in the membrane-attack complex/perforin family (MACPF) (Nagai et al. 2002; Oshiro et al. 2004; Satoh et al. 2007) were found only in *Cerebratulus* species. Previously identified nemertean peptide toxins, Neurotoxin B-II and Neurotoxin B-IV were not found, but we did not sequence the only species from which they are known (i.e., *C. lacteus*). Four species expressed the other known nemertean peptide toxin, Cytotoxin A-III.

**Table 3:** Toxin Genes Identified in Each Species

Species	Identifier	Trinotate Swiss-Prot annotation	Functional annotation and PFAM domains	Putative orthologs
*M. grossa*	M1	A0ZSK4: Neoverrucotoxin subunit beta	SNTX/VTX toxin; hemolytic activity	P1, Ch2, Cl1, Cm1, Llo1, Lr8
	M2	Q3SB03: Cysteine-rich venom protein pseudechetoxin-like	CAP domain; ShK domains; Potassium channel blocker	P2, Cl2
	M3	Q75WF2: Plancitoxin-1	Plancitoxin; DNase II domain; hepatotoxin	P4, T2, Ch3, Cl3, Cm2, Llo2, Lr2, Rl2
*Para. peregrina*	P1	A0ZSK3: Neoverrucotoxin subunit alpha	SNTX/VTX toxin; hemolytic activity	M1, Ch2, Cm1, Llo1, Lr8
	P2	Q3SB03: cysteine-rich venom protein pseudechetoxin-like	CAP domain; ShK domains; potassium channel blocker	M2, Cl2
	P3	Q98993: verrucotoxin subunit beta	SNTX/VTX toxin; hemolytic, cytolytic, hypotensive activities; calcium channel inhibitor	T1, Ch1, Cl1, Cm1, Llo1, Lr8, Rl1
	P4	Q75WF2: plancitoxin-1	Plancitoxin; DNase II domain; hepatotoxin	M3, T2, Ch3, Cl3, Cm2, Llo2, Lr2, Rl2
*T. polymorphus*	T1	Q91453: stonustoxin subunit beta	SNTX/VTX toxin; pore-forming; hemolytic, endema inducing activities	P3, Ch1, Cl1, Cm1, Rl1, Llo1
	T2	Q75WF2: plancitoxin-1	Plancitoxin; DNase II domain; hepatotoxin	M3, P4, Ch3, Cl3, Cm2, Llo2, Lr2, Rl2
	T3	Q76CA2: echotoxin-2	Echotoxin; anemone cytotox domain; pore-forming; hemolytic activity	Ch4, Cl4, Cm3, Lr3
	T4	Q66S13: natterin-4	Natterin; Aerolysin domain; edema and nociception induction	Cm6, Lla6, Lr7
*Cep. hongkongiensis*	Ch1	Q91453: stonustoxin subunit beta	SNTX/VTX toxin; pore-forming; hemolytic, endema inducing activities	P3, T1, Cl1, Lr1, Rl1
	Ch2	A0ZSK3: neoverrucotoxin subunit alpha	SNTX/VTX toxin; hemolytic activity	M1, P1, Cm1, Llo1, Lr8
	Ch3	Q75WF2: plancitoxin-1	Plancitoxin; DNase II domain; hepatotoxin	M3, P4, T2, Cl3, Cm2, Llo2, Lr2, Rl2
	Ch4	Q76CA2: echotoxin-2	Echotoxin; anemone cytotox domain; pore-forming; hemolytic activity	T3, Cm3, Lr3
	Ch5	B2DCR8: SE-cephalotoxin	Cephalotoxin; toxic function unknown	Cl5, Cm4, Lr4, Llo3, Rl3
	Ch6	P58912: toxin PsTX-60B	MACPF toxin domain; hemolytic activity	Cl6
	Ch7	Q76DT2: toxin AvTX-60A	MACPF toxin domain; hemolytic activity	Cl7
*Cep. linearis*	Cl1	Q91453: stonustoxin subunit beta	SNTX/VTX toxin; pore-forming; hemolytic, endema inducing activities	P3, T1, Ch1, Lr1, Rl1
	Cl2	Q2XXQ3: cystein-rich venom protein ENH1	CAP domain; ShK domains; potassium channel blocker	M2, P2
	Cl3	Q75WF2: plancitoxin-1	Plancitoxin; DNase II domain; hepatotoxin	M3, P4, T2, Ch3, Cm2, Llo2, Lr2, Rl2
	Cl4	Q76CA2: echotoxin-2	Echotoxin; anemone cytotox domain; pore-forming; hemolytic activity	T3, Ch4, Cm3, Lr3
	Cl5	B2DCR8: SE-cephalotoxin	Cephalotoxin; toxic function unknown	Ch5, Cm4, Llo3, Lr4, Rl3
	Cl6	P58912: toxin PsTX-60B	MACPF toxin domain; hemolytic activity	Ch6
	Cl7	Q76DT2: toxin AvTX-60A	MACPF toxin domain; hemolytic activity	Ch7
*C. marginatus*	Cm1	A0ZSK3: neoverrucotoxin subunit alpha	Neoverrucotoxin; hemolytic activity	P1, M1, Ch2, Llo1, Lr8
	Cm2	Q75WF2: plancitoxin-1	Plancitoxin; DNase II domain; hepatotoxin	P4, T2, M3, Ch3, Cl3, Llo2, Lr2, Rl2
	Cm3	Q76CA2: echotoxin-2	Echotoxin; pore-forming; hemolytic activity	T3, Ch4, Cl4, Lr3
	Cm4	B2DCR8: SE-cephalotoxin	Cephalotoxin; toxic function unknown	Ch5, Cl5, Llo3, Lr4, Rl3
	Cm5	P01527: cytotoxin A-III	Cytoxoxin A-III: pore-forming; kinase C, sodium, and calcium channel inhibitor	Ch5, Llo3, Lr4, Rl3
	Cm6	N/A: inferred as natterin-4 from reciprocal BLAST	Natterin; aerolysin domain; edema and nociception induction	T4, Lr7, Rl5
*L. longissimus*	Llo1	A0ZSK4: neoverrucotoxin subunit alpha	SNTX/VTX toxin; hemolytic activity	M1, P1, Ch2, Cm1, Lr8
	Llo2	Q75WF2: plancitoxin-1	Plancitoxin; DNase II domain; hepatotoxin	M3, P4, T2, Ch3, Cl3, Cm2, Lr2, Rl2
	Llo3	B2DCR8: SE-cephalotoxin	Cephalotoxin: toxic function unknown	Ch5, Cl5, Cm4, Lr4, Rl3
	Llo4	P01527: cytotoxin A-III	Cytoxoxin A-III: pore-forming; kinase C, sodium, and calcium channel inhibitor	Cm5, Llo4, Lr5, Rl4
*L. ruber*	Lr1	Q98989: stonustoxin	SNTX/VTX toxin; Pore-forming; Hemolytic, endema-inducing activities	P3, T1, Ch1, Cl1, Rl1
	Lr2	Q75WF2: Plancitoxin-1	Plancitoxin; DNase II domain; Hepatotoxin	M3, P4, T2, Ch3, Cl3, Cm2, Llo2, Rl2
	Lr3	Q76CA2: Echotoxin-2	Echotoxin; Pore-forming; Hemolytic activity	T3, Ch4, Cl4, Cm3
	Lr4	B2DCR8: SE-cephalotoxin	Cephalotoxin; toxic function unknown	Ch5, Cl5, Cm4, Llo3, Rl3
	Lr5	P01527: Cytotoxin A-III	Cytotoxin A-III; Pore-forming; Kinase C, sodium, and calcium channel inhibitor	Cl6, Llo4, Rl4
*R. lacteus*	Rl1	Q98989: stonustoxin	SNTX/VTX toxin; pore-forming; hemolytic, endema-inducing activities	T1, P3, Ch1, Cl1, Llo1
	Rl2	Q75WF2: plancitoxin-1	Plancitoxin; DNase II domain; hepatotoxin	M3, P4, T2, Ch3, Cl3, Cm2, Llo2, Lr2
	Rl3	B2DCR8: SE-cephalotoxin	Cephalotoxin: toxic function unknown	Ch5, Cl5, Cm4, Llo3, Lr4
	Rl4	P01527: cytotoxin A-III	Cytoxoxin A-III: pore-forming; kinase C, sodium, and calcium channel inhibitor	Lr4, Cm5, Llo3
	Rl5	Q66S13: natterin-4	Natterin; aerolysin domain; edema and nociception induction	T4, Cm6, Lr7

### Toxin Gene Trees

The SNTX dataset had 33 sequences and 577 aligned amino acid positions; the best tree had a likelihood value of −17,869.9071 (supplementary fig. S2, Supplementary Material online). The Plancitoxin/DNase II dataset had 23 sequences and 442 aligned amino acid positions; the best tree had a likelihood value of −10,952.2682 (supplementary fig. S3, Supplementary Material online). Both datasets have been deposited on figshare (doi:10.6084/m9.figshare.1203580). Nemertean SNTX-like genes were all more closely related to the recently described SNTX-like genes from *Glycera* polychaetes than stonefish genes. A large clade of all nemertean SNTX-like transcripts and *Glycera* SNTX genes were sister to toxins from stonefish. Plancitoxin-1 like genes were more closely related to Plancitoxin-1 from *Acanthaster planci*—the species from which it was described—but nodal support values were so low for other relationships that they should be approached with caution.

## Discussion

### Nemertean Toxins

Nemerteans have long been known to use toxins in predation and defense (Kem 1971; Ali et al. 1990; Heine et al. 1991; McClintock and Baker 1997; Asakawa et al. 2013), but our findings indicate a previously unidentified diversity of putative toxin genes among members of the phylum (table 3). Prior studies of nemertean toxins have focused primarily on *P. corrugatus* or *Cerebratulus* spp. and only identified three protein toxins (Kem 1976, 1994; McClintock and Baker 1997; Berne et al. 2003). In contrast, annotation of nemertean transcriptomes found expression of multiple putative toxin genes, of which only one was previously known from nemerteans. Differences in the presence of specific toxin genes hint that their expression may depend on the life habits and/or evolutionary history of each species, particularly for the commensal *M. grossa*, which had the fewest observed toxin genes. However, other factors such as active or resting state of venom glands when each animal was sacrificed, gender, or physiological state could also be responsible for such differences (Morgenstern et al. 2011; Menezes et al. 2006).

Plancitoxin-1, a protein with homologs found in all nine nemerteans analyzed here and one of the few known toxic DNase II proteins (Shiomi et al. 1990, 2004; Ota et al. 2006), is a hepatotoxin found in the crown-of-thorn starfish (*A. planci*). *Acanthaster* Plancitoxin-I is inactive below pH 5.5 and most active at pH 7.2 (Shiomi et al. 2004), but mammalian DNase II proteins function best at pH 5.0 (Counis and Torriglia 2000; Evans and Arguilera 2003). Some nemerteans are known to have acidic body walls (e.g., *P. corrugatus*, *Cephalothrix spiralis*, *Lineus socialis*; Ferraris 1979; Heine et al. 1991), and putatively toxic DNase II proteins in nemerteans may function well at low pH levels like mammalian DNase II proteins. As such, nemerteans that secrete acidic mucus may do so to improve the effect of toxin peptides, rather than as a standalone defensive mechanism.

Genes with high similarity to toxins in the SNTX/VTX protein family were also found in all nine nemerteans analyzed. SNTX/VTX toxins have cytolytic properties and were found previously in fish (Ueda et al. 2006), monotremes (Wong et al. 2013), and *Glycera* annelids (von Reumont et al. 2014). These toxins are utilized for defense, sexual competition, and predation in fish, monotremes, and *Glycera*, respectively. Because whole animals, rather than only epidermis or proboscis-associated glands, were used for most RNA extractions here, tissue-specific isolation of SNTX/VTX toxins will be necessary to determine if such toxins are used by nemerteans for defense, predation, or both.

Of the three previously identified nemertean peptide toxins, we only found transcripts for Cytotoxin A-III-like proteins (Kem 1994; Berne et al. 2003). This may indicate that Neurotoxin B-II and Neurotoxin B-IV are not commonly expressed in many nemerteans. Cytotoxin A-III has been found in the epidermis of some nemerteans, where it serves as a defensive neurotoxin (Kem and Blumenthal 1978; Berne et al. 2003). This toxin was initially described from the heteronemertean *C. lacteus*. As expected, it was also present in the closely related *C. marginatus*. Furthermore, both *Lineus* species and *R. lacteus*, all three of which are also heteronemerteans (Sundberg et al. 2001; Thollesson and Norenburg 2003; Kvist et al. 2014), also expressed Cytotoxin A-III-like genes.

In contrast to Cytoyoxin A-III being present only in heteronemerteans, evolutionary relatedness alone does not appear to explain which species were found to express SE-cephalotoxin-, Echotoxin-2-, and Natterin-4-like genes. For example, SE-cephalotoxin-like genes were expressed in four heteronemerteans and the paleonemetean *Cep. hongkongiensis* but were not found in the remaining species. However, this could be a result of different expression patterns rather than SE-cephalotoxin-like genes being absent from the genomes of the other four species. Presence or absence of Echotoxin-2- and Natterin-4-like genes was also similar to that of SE-cephalotoxin-like genes. Interestingly, SE-cephalotoxin and natterins have previously been found only in cephalopods and fish, respectively (Tamura et al. 2011; Barathkuma et al. 2013). SE-cephalotoxin is found in salivary glands of the cuttlefish *Sepia esculenta* and is utilized in predation, but its precise biochemical function is unknown (Ueda et al. 2008). On the other hand, natterins from fish all have aerolysin domains and induce edema and nociception (Xue et al. 2012). Nemertean Natterin-4-like genes also possess an aerolysin domain, which is suggestive of a similar function.

While ShK toxins and their characteristic domains were initially described from the sea anemone *Stichodactyla helianthus* (Castañeda et al. 1995), they have also been found in snake venom (Shikamoto et al. 2005; Wang et al. 2006) and *Glycera* annelids (von Reumont et al. 2014). Interestingly, ShK toxin-like genes identified here were more similar at the amino acid level to ShK toxin genes in snakes (table 3) with whom nemerteans share a more recent common ancestor than sea anemones. ShK domains in nemertean ShK toxin-like proteins were always downstream of cysteine-rich secretory domains, which have toxic properties in other invertebrates (e.g., in Hymenoptera; Lu et al. 1993) further indicating toxic properties of these peptides. Because ShK toxins function as potassium-channel blockers, they have been proposed as human pharmaceutical targets to treat obesity (Upadhyay et al. 2013), graft rejection, and autoimmune diseases (Kalman et al. 1998). Thus, nemertean ShK-like toxins may have applicability in future drugs.

MACPF proteins are found in bacteria and many eukaryotes where they function in innate immunity (Young et al. 1986; Morito-Yamamuro et al. 2005; Tosi, 2005), pathogenesis (Kaiser et al. 2004), or less commonly, in predatory/prey interactions (Oshiro et al. 2004; Satoh et al. 2007). Given such a wide utility, it is not surprising that we found genes encoding MACPF protein domains. Genes coding for MACPF proteins in both *Cephalothrix* species had high sequence similarity at the amino acid level to toxin genes found in the sea anemone *Actineria villosa* (Oshiro et al. 2004) and *Phyllodiscus semoni* (Satoh et al. 2007). This suggests that some nemerteans may use MACPF toxins in defense and/or prey capture in an analogous fashion as sea anemones.

### Nemertean Transcriptome Characterization

Fewer than half of all contigs from assemblies of each species were annotated with ORFs or other annotations, but of those contigs with predicted ORFs over 60% in each species were annotated with a Swiss-Prot hit (table 2). Unannotated contigs likely result from a combination of incompletely assembled transcripts, noncoding regions, and proteins of unknown function. Notably, the percentage of contigs with predicted ORFs ranged from 18.2% in *C. marginatus* to 46.2% in *T. polymorphus*. In contrast, the percentage of ORFs with Swiss-Prot annotation hits for each species was higher and more uniform (63.1–74.4%; table 2). This indicates the presence of a group of nemertean proteins with unknown function—some of which could be toxins. In addition, uniformities in the percentage of genes classified for higher level GO terms among all taxa provide evidence of conserved higher-level function of many nemertean genes and conserved cellular physiology in nemerteans (fig. 1*A*–*G*). Such characterizations fail to capture similarities or differences in unannotated genes, but they highlight the general homogeneity of transcriptome function among nemerteans. For example, nearly half (44–48%) of all annotated ORFs in each species were categorized as biological processes, and differences among species for the other two higher-level GO terms varied by not more than 2%. Percentages of genes annotated for lower-level GO terms were more varied (fig. 1), which provides evidence of some transcriptome specialization and/or differences in gene expression among nemertean lineages. Despite advances in sequencing technologies, functional work is still needed to characterize the large fraction of transcripts and putative proteins of unknown function.

## Conclusions

Transcriptome analysis of nine nemertean species uncovered nine different types of toxin-like genes—eight of which were previously unknown from nemerteans. Nemerteans are one of three animal lineages known to utilize toxins for both predation and defense (Casewell et al. 2013), and peptide toxins clearly contribute to the overall toxin suite in ribbon worms. Two recent reviews emphasized convergent evolution of predatory and defensive toxins in animals (Fry, Roelants, Champagne, et al. 2009; Casewell et al. 2103). Furthermore, Fry, Roelants and Norman (2009) demonstrated that toxin genes in cephalopods resemble toxins in other lineages and concluded that peptide toxins have evolved from nontoxic proteins that are found across animals. These studies did not include nemerteans, but similarties among putative nemertean toxins and toxin proteins in disparate lineages (e.g., cnidarians, molluscs, fish) indicate that convergent evolution of toxins from nontoxic proteins may have also occurred in nemerteans. Identifying putative nemertean toxins here relied on sequence similarities to well-known, annotated animal toxins, and many more toxins that are unique to nemerteans may await discovery. Sequence and structural similarity to peptide toxins in other organisms is evidence of shared function, but experimental studies are needed to understand the precise function of putative toxin genes identified here and to explore how different toxins work together to provide defense from predators or enhance predatory behaviors.

## Supplementary Material

Supplementary table S1, figures S1–3 are available at *Genome Biology and Evolution* online (http://www.gbe.oxfordjournals.org/).

Supplementary Data
