# Supplementary material for: Nemertean Toxin Genes Revealed through Transcriptome Sequencing
Source: Genome Biol Evol. 2014 Nov 27;6(12):3314–25. doi: 10.1093/gbe/evu258 (PMC4986456; doi:10.1093/gbe/evu258)
Supplement: Supplementary Data [file supp_6_12_3314__index.html]

Nemertean Toxin Genes Revealed Through Transcriptome Sequencing — Nemertean Toxin Genes Revealed through Transcriptome Sequencing — Supplementary Data 

# Nemertean Toxin Genes Revealed through Transcriptome Sequencing

## Supplementary Data

files

**Files in this Data Supplement:**

- Supplementary Data - doc file
