# Supplementary material for: Nemertean Toxin Genes Revealed through Transcriptome Sequencing
Source: Genome Biol Evol. 2014 Nov 27;6(12):3314–25. doi: 10.1093/gbe/evu258 (PMC4986456; doi:10.1093/gbe/evu258)
Supplement: Supplementary Data [file supp_evu258_NemerteanToxinsSupMat.doc]

**Supplementary Material**

fig. s1.


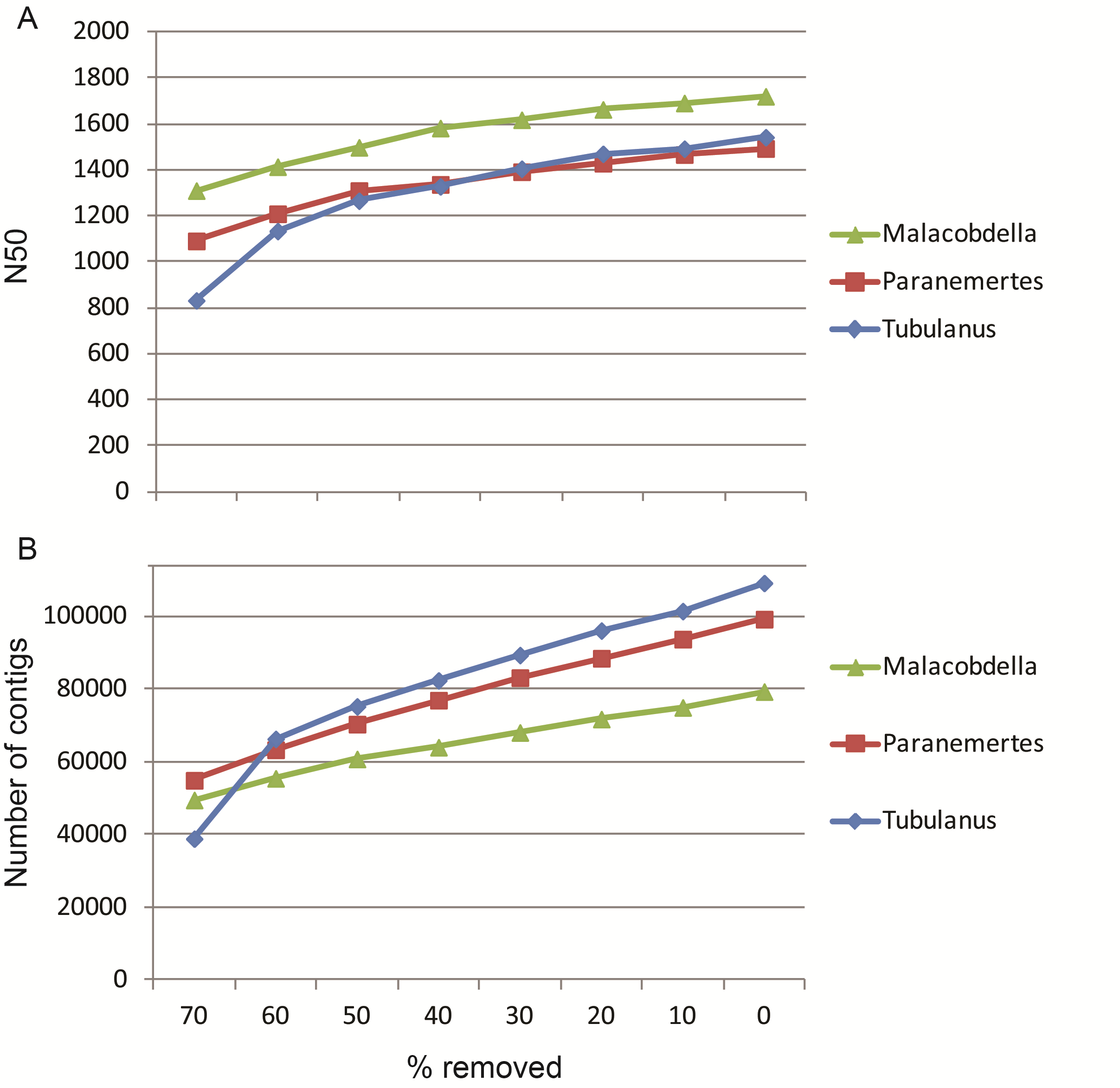


fig. s2.


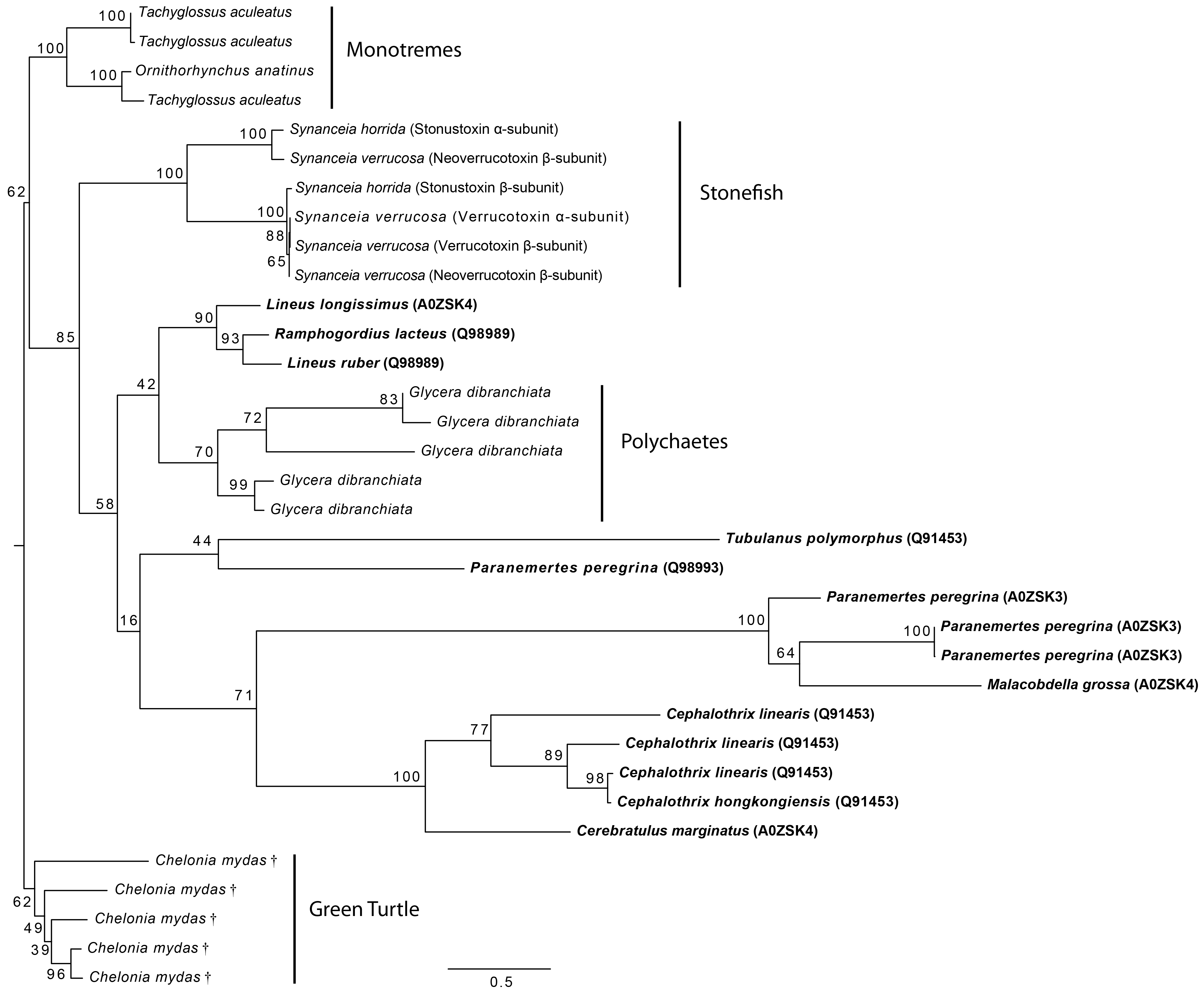


fig. s3.


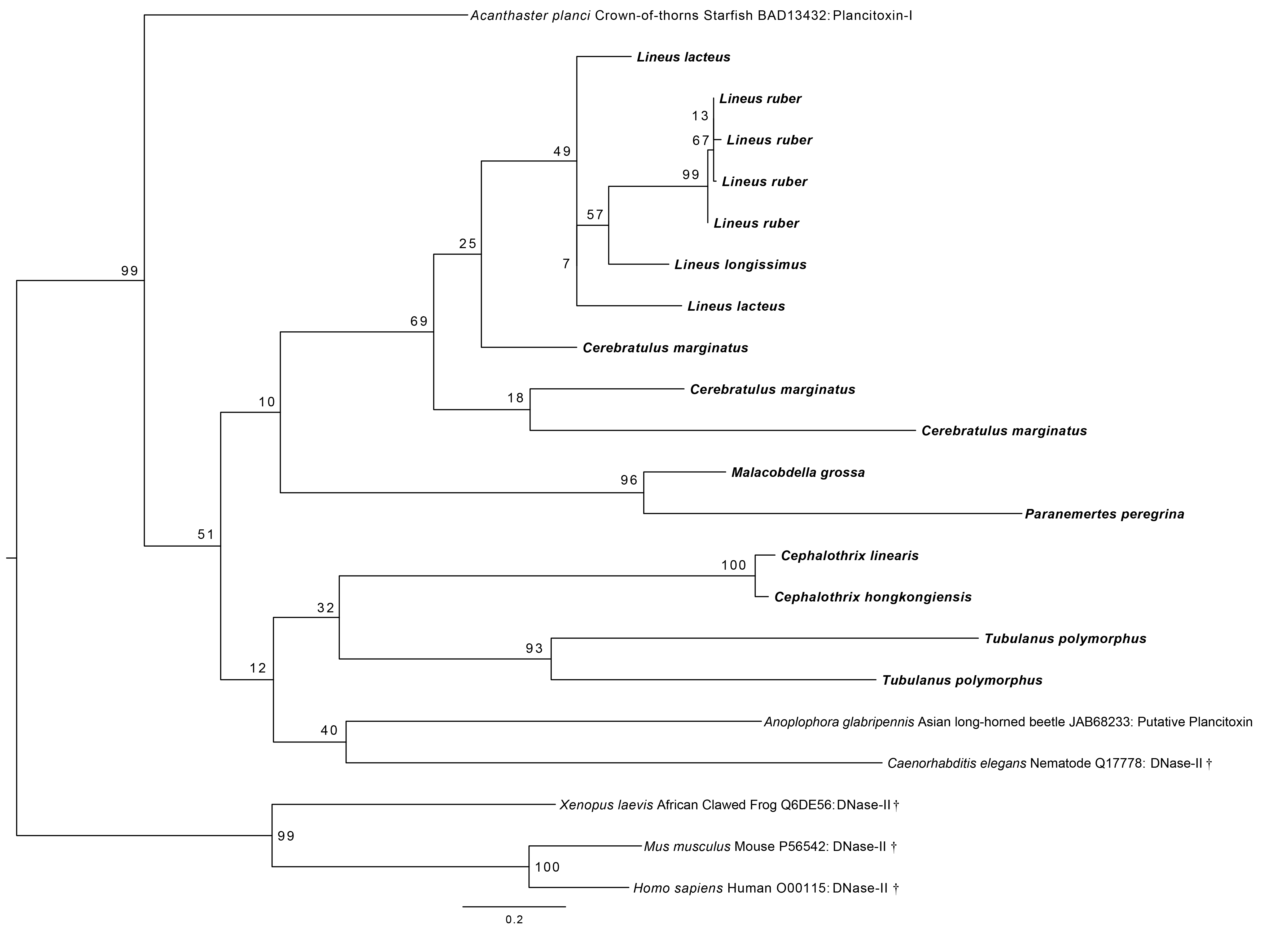


Supplementary Figure Legends

fig. s1. Rarefaction curves of assembly statistics for the three nemerteans sequenced here. A) N50 B) Contig length.

fig. s2. Maximum likelihood SNTX gene tree. Nemertean transcripts are in bold and initial Trinotate annotations for each transcript are in parentheses. Non-nemertean sequences are from von Reumont et al. (2014), and the tree is rooted with *Chelonia mydas* as in von Reumont et al. (2014). †: Non-toxic genes.

fig. s3. Maximum likelihood Plancitoxin-1 gene tree. Nemertean transcripts are in bold. All nemertean transcripts were identified as Plancitoxin-1 by Trinotate. Non-nemertean sequences are labeled with their uniprot or genbank identifiers. The tree is rooted with non-toxic DNase II genes. †: Non-toxic genes.
